# Interface-Coupling of NiFe-LDH on Exfoliated Black Phosphorus for the High-Performance Electrocatalytic Oxygen Evolution Reaction

**DOI:** 10.3389/fchem.2022.951639

**Published:** 2022-07-07

**Authors:** Jinchen Fan, Xi Qin, Wendan Jiang, Xiaolei Lu, Xueling Song, Wenyao Guo, Sheng Zhu

**Affiliations:** ^1^ School of Materials and Chemistry, University of Shanghai for Science and Technology, Shanghai, China; ^2^ Shanghai Key Laboratory of Materials Protection and Advanced Materials in Electric Power, College of Environmental and Chemical Engineering, Shanghai University of Electric Power, Shanghai, China

**Keywords:** NiFe LDH, black phosphorus, interface-coupling, charge transfer, oxygen evolution reaction

## Abstract

Electrochemical oxygen evolution reaction (OER) always plays an important role in many electrochemical energy storage and conversion systems. Owing to the slow kinetics mainly brought from multiple proton-coupled electron transfer steps, the design and exploit low-cost, highly active, durable OER electrocatalysts are of significant importance. Although the black phosphorus (BP) shows good electrocatalytic OER performance, it still faces the problems of poor intrinsic activity and low stability due to its instability under ambient conditions. The NiFe-LDH was assembled onto the surfaces of exfoliated BP (EBP) nanoflakes to realize the interfacial coupling between them, achieving an effective improvement in electrocatalytic activity and stability. Benefitting from the interfacial P-O bonding, the NiFe-LDH@EBP hybrid shows high OER activity with a low overpotential of ∼240 mV@10 mA cm^−2^ toward OER under alkaline conditions, as well as the good stability. Density functional theory (DFT) calculations proved that the interface-coupling of NiFe-LDH on BP promotes charge transfer kinetics and balances the adsorption/desorption of reaction intermediates, ultimately imparting excellent OER electrocatalytic activity.

## GRAPHICAL ABSTRACT

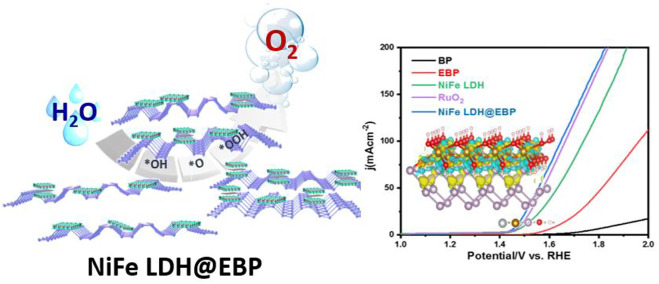

## Introduction

Hydrogen (H_2_) produced by electrochemical water decomposition is always considered to be one of the promising solutions to the problem of fossil fuel depletion (Suen et al., 2017; Tian et al., 2019). Electrochemical water splitting generally involves two half-reactions, hydrogen evolution reaction (HER), and oxygen evolution reaction (OER) (Song et al., 2020). Its overall reaction efficiency is often limited by the slow kinetics of OER. As the important half-reaction, the high-performance catalyst is one of the keys to high-efficient OER. In common, noble metal electrocatalysts for OER such as Ru/Ir-based compounds have been widely used for water splitting, but the resource scarcity and high cost significantly hinder their further industrial application. In this case, high-performance and low-cost non-precious metal or metal-free catalysts become the direction of design and research toward electrocatalytic OER (Stoerzinger et al., 2014; Mo et al., 2018).

As a kind of fascinating two-dimensional nanomaterial, black phosphorus (BP) has been considered a promising electrocatalyst for OER due to its conductivity, chemically reactivity, and band gap tunability (Shen et al., 2015; Qiu et al., 2017; Ren et al., 2017; Liu et al., 2018). For the first time, Wang et al. (2014) prepared the BP–Ti thin film and BP–carbon nanotube hybrid and innovatively demonstrated that BP can well drive the oxygen evolution reaction (OER). For the van der Waals layered structure of BP, the exfoliation and thickness reduction could not only effectively improve the electrical transport performance but also increase the number of active sites and improve the OER performance of BP (Huang et al., 2019; Lee et al., 2021). Ren et al. (Ren et al., 2017) prepared the few-layered BP nanosheets by liquid-phase exfoliation and found that the OER onset-potential and Tafel slope of BP nanosheets were achieved at 1.45 V and 88 mV dec^−1^. Although the BP and exfoliated BP nanosheets show good electrocatalytic OER performance, the BP itself still faces the problems of poor intrinsic activity and low stability due to its instability under ambient conditions (Wang et al., 2014; Zhu et al., 2017; Wang et al., 2018). To solve the aforementioned problems, the methods of surface modification, heterostructure construction, and heteroatom-doping, etc., were used to enhance the OER performances of BP (Guo et al., 2015; Bat-Erdene et al., 2017; Liu et al., 2019; Liu et al., 2022). Wang et al. (2018) prepared *in-plane* BP/Co_2_P heterostructures *via in situ* reduction of Co at BP defect/edge sites. The BP/Co_2_P nanosheets showed highly stable and excellent HER and OER performance even at large potential and current densities. Qiao et al. (2020) also reported the hybrid dimensional BP/Au catalyst with Au nanoparticles (NPs) supported on BP nanosheets (BP/Au) which was synthesized by a one-pot solution-phase method. Thence, based on the characteristics of the two-dimensional layered structure, the defects of BP nanosheets could be used to construct the supported OER catalyst for improving stability and performance (Tan et al., 2017).

Always, layered double hydroxides (LDHs) are known as anionic layered materials, mainly composed of cationic layers of metal hydroxides and charge-balancing anions mixed in the layer and layer regions (Wang and O’Hare, 2012). Various LDHs have been shown to be potential electrocatalysts for promoting the oxygen evolution reaction (OER) due to their inherent high activity and large specific surface area (Jamesh and Harb, 2021). Among the different LDH catalysts, NiFe-LDH often shows relatively good activity and stability under alkaline conditions, making it become the research hotspot of OER under alkaline conditions (Liu et al., 2020). Although NiFe-LDH has shown great promise in OER electrocatalysis, the catalytic sites of NiFe-LDH are only limited to some specific sites, and the conductivity and stability of the NiFe-LDH are still defective (Luo et al., 2017; Bi et al., 2018; Peng et al., 2021). To improve the conductivity and stability of NiFe-LDH toward OER, lots of conductive supports including multi-walled carbon nanotubes (CNTs), graphene nanosheets, Ni foam, and 3D macro/mesoporous carbon played as the support of the NiFe-LDH which could jointly active the sites of NiFe-LDH and synergistically enhance the OER performance (Jiang et al., 2013; Tonda et al., 2018; Wang et al., 2019).

Based on the previously mentioned analysis, we prepared the NiFe LDH@ exfoliated BP nanoflakes with NiFe-LDH supported on the surfaces of BP through interface-coupling via P-O bonding. The synergistic interaction between BP and NiFe LDH not only accelerated the electron/mass transfer but also improved the adsorption/desorption of reaction intermediates. The NiFe LDH@EBP hybrids exhibit a high overpotential of ∼240 mV at a current density of 10 mA cm^−2^ which is higher than that of NiFe LDH and EBP. Meanwhile, the NiFe LDH@EBP also shows catalytic durability with ∼99.1% current density retention.

## Experimental Section

### Exfoliation of Bulk Black Phosphorus

The exfoliated black phosphorus (EBP) nanoflakes were exfoliated from the bulk BP via liquid-phase exfoliation. Briefly, 50 mg of BP was ground into a powder in an Ar-filled glove box and then transferred to 50 ml of dehydrated and Ar-saturated N-methylpyrrolidone solvent. After continuous sonication for 12 h in an ice-water bath, the EBP was collected with centrifugation at a speed of 15,000 rpm for 20 min and washed with lots of dehydrated ethanol followed with drying naturally.

### Synthesis of NiFe-LDH

NiFe-LDH was prepared by a co-precipitation method. First, 0.017 mol of Fe(NO_3_)_3_·6H_2_O and 0.034 mol of Ni(NO_3_)_2_·6H_2_O were dissolved in 50 ml of deionized water as precursor solution. Then, under vigorous stirring, 50 ml of aqueous solution containing 0.117 mol of NaOH and Na_2_CO_3_ was added dropwise to the previous precursor solution. After adjusting the pH value to 9.5 using 1 M nitric acid solution, the resulting slurry was stirred at room temperature for 2 h. Finally, the suspension was stirred at 50°C for 2 days to complete the reaction. The obtained NiFe-LDH precipitate was collected by filtration, washed with deionized water, and dried overnight at 80°C.

### Preparation of the NiFe LDH@EBP Hybrids

The NiFe LDH@EBP hybrid was prepared by the co-assembly method. Desired amounts of NiFe-LDH and EBP were added into 25 ml of dimethylsulfoxide followed by bath sonication for 4 h and then left to stand for 2 h at room temperature. Next, the LDH@EBP hybrids were collected by centrifugation at a speed of 8,000 rpm for 10 min and vacuum-dried at 60^o^C for 12 h. By changing the feed weight ratio of NiFe-LDH to EBP, a series of NiFe LDH@EBP hybrids of NiFe LDH@EBP (1:8), NiFe LDH@EBP (1:4), NiFe LDH@EBP (1:2), and NiFe LDH@EBP (1:1) were obtained.

### Instruments and Characterization

X-ray diffraction (XRD) patterns of NiFe-LDH, EBP, and NiFe LDH@EBP were recorded on a Bruker AXS D8 Advance diffractometer with Cu Kα radiation (*λ* = 1.541874 Å). A field emission transmission electron microscope (JEM-2100F) was used to characterize the morphologies of NiFe-LDH, EBP, and NiFe LDH@EBP. Raman spectra of NiFe-LDH, EBP, and NiFe LDH@EBP were collected on a Lab RAM HR800 Raman spectrometer (HORIBA Jobin Yvon, France). The X-ray photoelectron spectra (XPS) of NiFe-LDH, EBP, and NiFe LDH@EBP were obtained using a Thermo Scientific ESCALAB 250Xi XPS using Al Kα radiation.

### Electrocatalytic Measurement

The electrocatalytic OER performance of NiFe LDH@EBP was measured by using a rotating disc electrode (RDE) (Pine Research Instrumentation) system. The catalyst-coated glassy carbon RDE, Hg/HgO, and graphite rod electrodes were used as a working electrode, reference electrode, and counter electrode, respectively. The catalyst coating on the RDE was performed by dropping 10 μl of catalyst ink and then allowed drying. The conversion between potential to RHE and to Hg/HgO electrodes was performed using the following equation conversion: E (RHE) = E (Hg/HgO) + 0.0591×pH + 0.098. LSV and stability tests were performed on the O_2_-saturated KOH electrolyte (1 M) with a scan rate of 50 mV/s and *iR* compensation of 95%. The Tafel slope was fitted by the LSV curve with the equation *η = a + b × log |j|*, where *j* is the current density, *b* is the Tafel slope, and *η* is the overpotential. The ECSA of the catalyst is calculated using the double-layer capacitance (Cdl). The CV test is performed at scan rates from 10 to 100 mV/s.

## Results and Discussion

**FIGURE 1:**
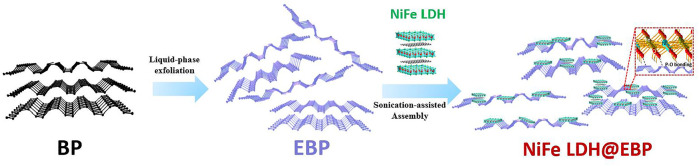
Schematic illustration for the preparation of NiFe LDH@EBP.

**FIGURE 2:**
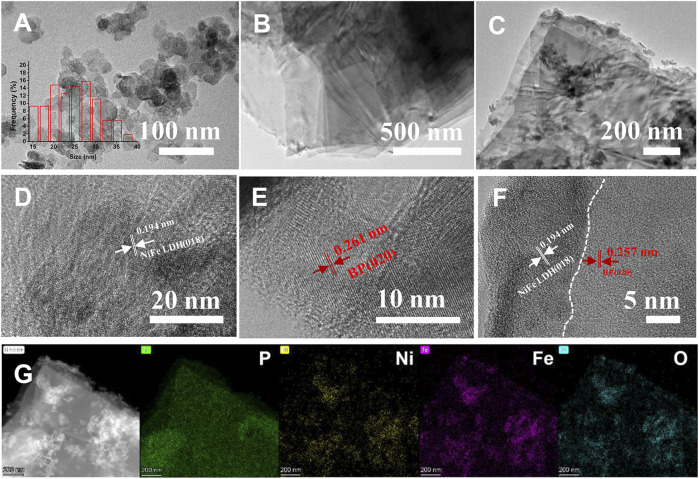
**(A)** TEM image of NiFe-LDH (inset is the histogram of lateral size distribution), **(B)** high-resolution transmission electron microscope (HRTEM) image of NiFe LDH, **(C)** TEM image of EBP nanoflakes, **(D)** HRTEM image of EBP, **(E)** TEM image of NiFe LDH@EBP, **(F)** HRTEM image of NiFe LDH@EBP and its corresponded distance of crystal planes, and **(G)** HAADF-STEM image and EDS element mapping images of P, Ni, Fe, and elements.

As shown in Figure 1, the hybrid catalyst of NiFe LDH@EBP was prepared via the electrostatic co-assembly method with the assistance of sonication. During the sonicating process, the NiFe-LDH was exfoliated into thin NiFe LDH flakes accompanied by a size decrease. The exfoliated NiFe-LDH could be assembled onto the surfaces of EBP flakes of relatively larger size through electrostatic adsorption. Meanwhile, the highly reactive phosphorus atoms of EBP can also react with the surface oxygen of NiFe-LDH to form the P-O valence bonds, thus facilitating the anchoring of NiFe-LDH on EBP. The microstructure and morphologies of the NiFe LDH@EBP were first characterized by transmission electron microscopy (TEM) images. As illustrated in Figure 2A, the exfoliated NiFe LDH exhibits distinct round thin flakes with a mean size of ∼25.6 nm. The lattice spacing of 0.194 nm corresponds to the (018) crystal plane of NiFe-LDH (Figure 2B). Toward the pristine EBP flakes as shown in Figure 2C, the layered structure with a horizontal size of several micrometers could be easily observed, and their surfaces seem to be very smooth and clean. From the HRTEM image (Figure 2D), the EBP shows good crystallinity with distinct lattice fringes. The lattice spacing of 0.261 nm is attributed to the (020) crystal plane of BP. It can be seen from Figure 2E that after hybridization with small-size NiFe-LDH, the surfaces of EBP become rough and have been modified by NiFe-LDH nanoparticles. As illustrated in Figure 2F, the spacings of 0.194 and 0.257 nm were ascribed to the (018) crystal plane of NiFe-LDH and (020) crystal plane of BP, demonstrating the combination of NiFe-LDH and EBP. By observing the high-resolution high-angle annular dark-field (HAADF) scanning transmission electron microscopy (STEM) image (Figure 2G), we found that the small-size NiFe-LDH particles reflected with bright white color were densely decorated on the surfaces of EBP (Gusmão et al., 2017; Ren et al., 2017). From the STEM-EDS mapping images, the distributions of P, Ni, Fe, and O elements proved the formation of NiFe LDH@EBP hybrid catalyst.

**FIGURE 3:**
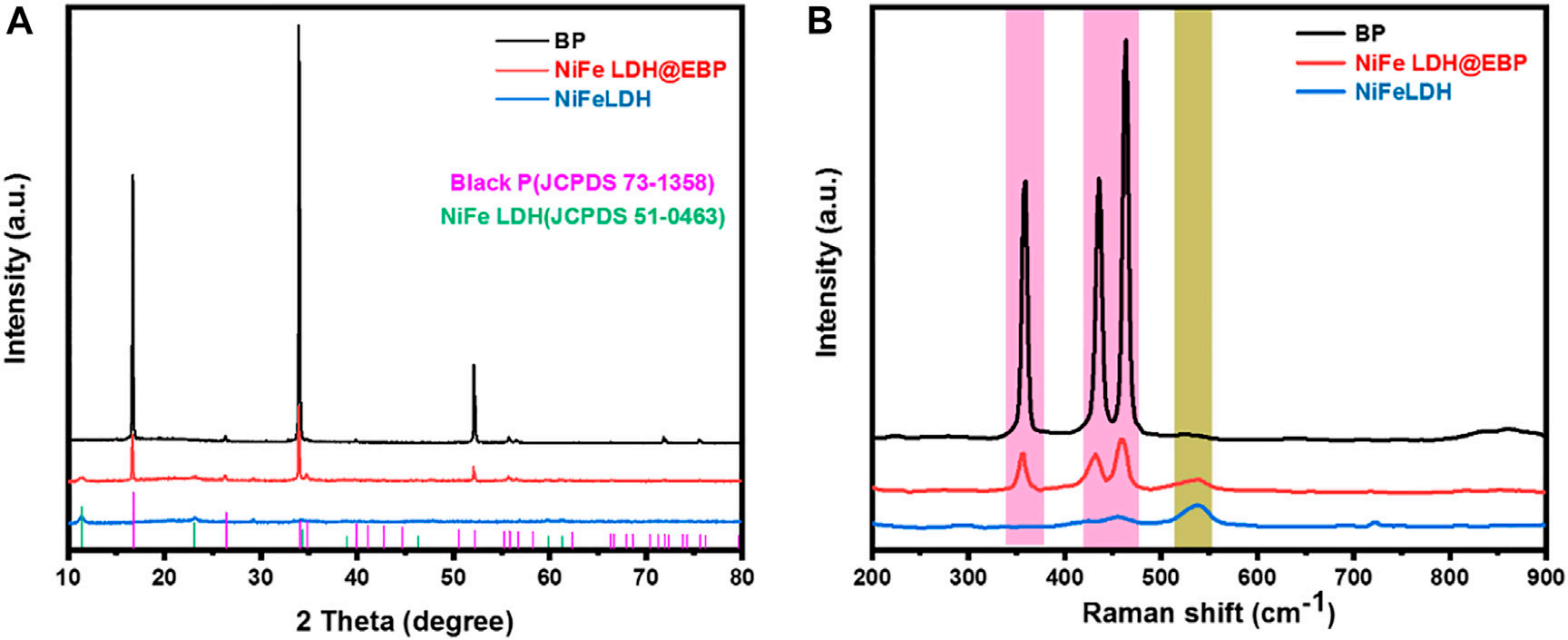
**(A)** XRD patterns for BP, NiFe-LDH, and NiFe LDH@EBP; **(B)** Raman spectra of BP, NiFe-LDH, and NiFe LDH@EBP.

As shown in Figure 3A, three strong crystallization peaks in the XRD patterns of BP that appear at 2θ = 16.9°, 34.2°, and 52°, respectively, are attributed to the (020), (040), and (060) planes of orthorhombic BP which matches well with the standard pattern for BP (JCPDS Card No. 73-1358)(Wang et al., 2015; Gusmão et al., 2017). The characteristic diffraction peaks of (003), (012), and (110) for NiFe-LDH were located at 2θ values of 11.9°, 23.1°, and 38.9°, respectively, which is consistent with the standard pattern for NiFe LDH (JCPDS Card No. 51-0463) (Yang et al., 2017). Toward the NiFe LDH@EBP hybrid, there are no new diffraction peaks, and the characteristic diffraction peaks of NiFe-LDH and BP all exist, demonstrating the combination of NiFe-LDH, and EBP (Iglesias et al., 2005; Liu et al., 2017; Zhang et al., 2018b; Wang et al., 2021). The Raman spectra for the NiFe-LDH, EBP, and NiFe LDH@EBP were further analyzed. As shown in Figure 3B, there are three typical Raman peaks at 360.5, 435.6, and 463.8 cm^−1^, which can be assigned to A_g_^1^, B_2g_, and A_g_^2^ phonon modes of BP, respectively. The Raman peaks of NiFe LDH were observed at Raman shifts of 465, 542, and 726 cm^−1^. After incorporating into EBP, in the Raman spectrum of NiFe LDH@EBP hybrid, the Raman peak around 542 cm^−1^ is originated from NiFe-LDH, and the typical Raman peaks of BP show the evident slight blue-shift, revealing the interaction between NiFe-LDH and EBP (Li et al., 2017; Zhang et al., 2022).

**FIGURE 4:**
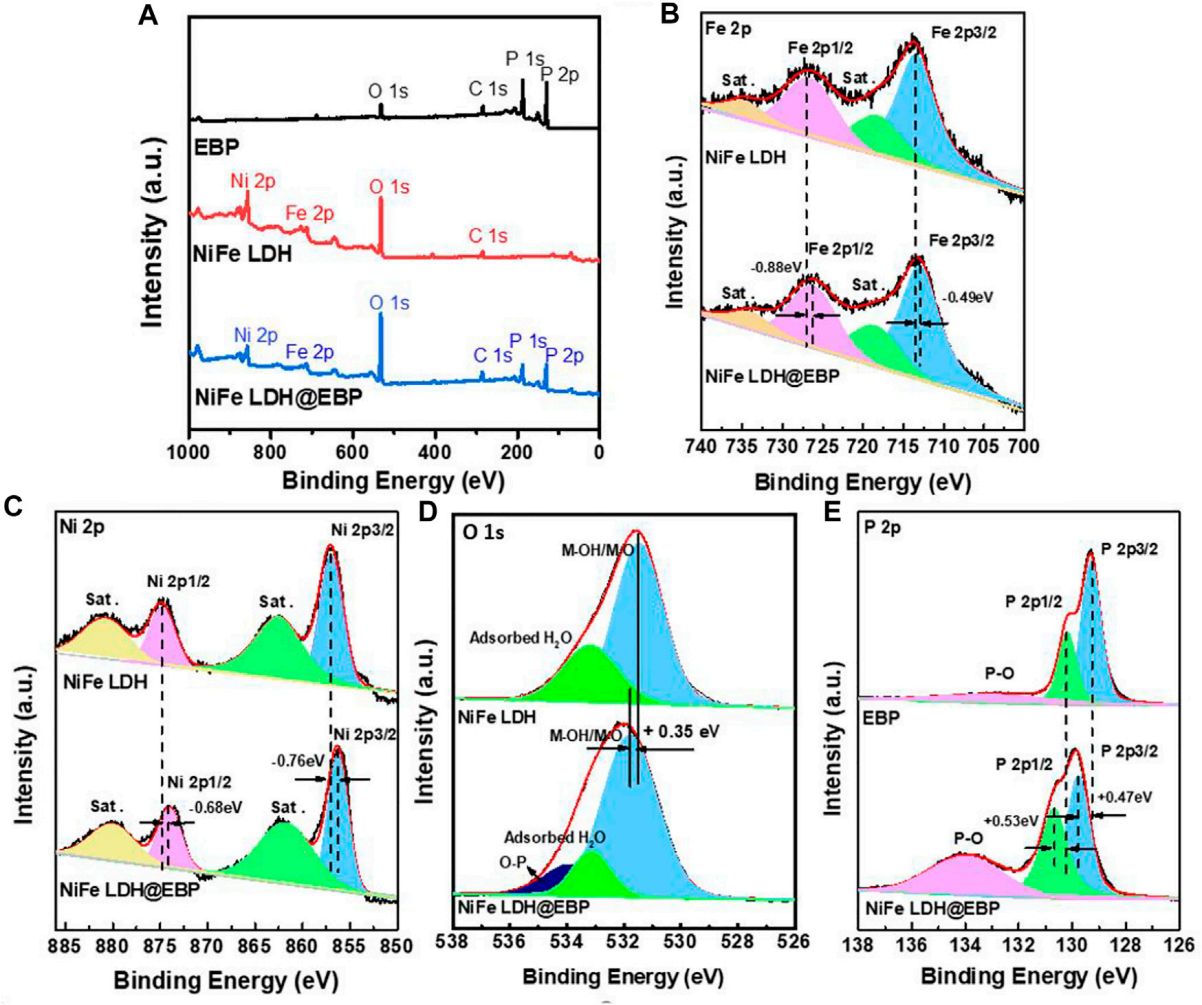
**(A)** XPS survey spectra for EBP, NiFe-LDH, and NiFe LDH@EBP, high-resolution XPS spectra for **(B)** Fe 2p, **(C)** Ni 2p, and **(D)** O 1s of NiFe-LDH and NiFe LDH@EBP, and **(E)** P 2p core-level spectra of EBP and NiFe LDH@EBP.

To further understand the combination of NiFe-LDH and EBP, XPS spectra were used to characterize the valences and compositions of NiFe LDH@EBP. From the XPS survey spectrum of NiFe LDH@EBP (Figure 4A), the existence of four elements of P, Ni, Fe, and O also implies the combination of NiFe-LDH and EBP which is consistent with the analysis of STEM-EDS mapping images. In Figures 4B,C, there are two shake-up satellites in both the high-resolution Ni 2p and Fe 2p spectra. For the core-level Ni 2p spectrum, the peaks at 854.7 and 874.8 eV are ascribed to the two spin-orbit peaks of Ni 2p_3/2_ and 2p_1/2_ of Ni^2+^, respectively (Zhang. et al., 2018a; Li et al., 2020). After assembled on the surfaces of EBP, the binding energies were both shifted to higher energies demonstrating the strong interaction between NiFe-LDH and EBP support. Similarly, the peaks for Fe 2p_1/2_ and Fe 2p_3/2_ were located at 727.1 and 713.6 eV. Compared to the Fe 2p spectrum of NiFe LDH@EBP, there are also shifts to lower binding energies for the peaks of Fe 2p_1/2_ and Fe 2p_3/2_ induced by the interaction between NiFe-LDH and EBP. Toward the O 1s spectrum of NiFe-LDH illustrated in Figure 4D, the peaks at 533.2 and 531.6 eV were attributed to the oxygen of adsorbed H_2_O and M–O/M–OH (M = Ni or Fe), respectively. In comparison, there is a new peak at 533.9, implying the generation of P-O bonding. Also, in the P 2p spectrum (Figure 4E), the peak intensity and area of P-O bonding appeared at 134.1 eV increased after incorporating into NiFe-LDH. Meanwhile, there are evident positive shifts of 0.35, 0.47, and 0.53 eV for M–O/M–OH (M = Ni or Fe) which may also come from the interaction of NiFe-LDH and EBP.

**FIGURE 5:**
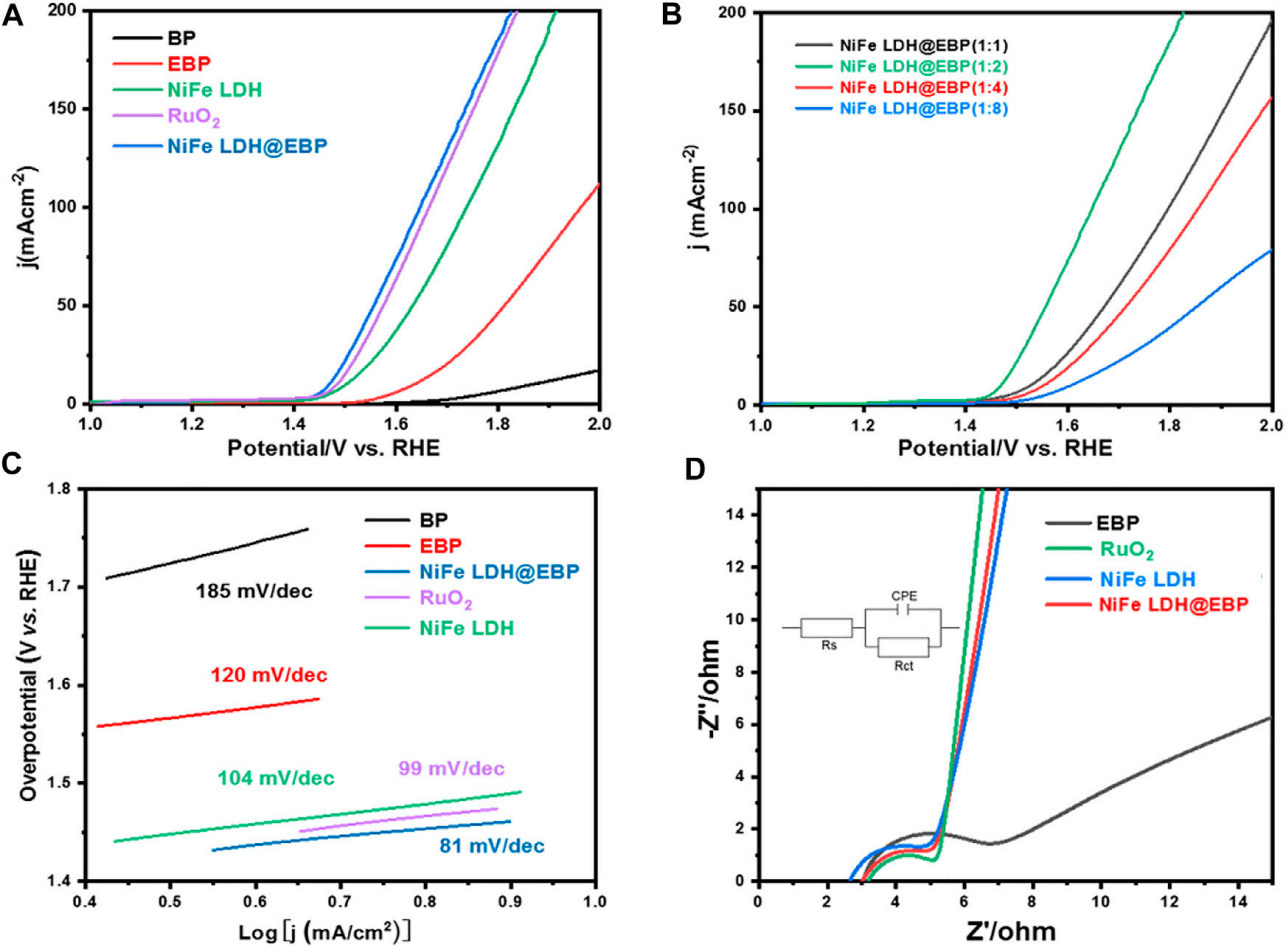
**(A)** LSV curves for BP, EBP, NiFe-LDH and NiFe LDH@EBP, and RuO_2_, **(B)** LSV curves of NiFe LDH@EBP samples with different feed weight ratios of NiFe-LDH to EBP, **(C)** Tafel plots for BP, EBP, NiFe-LDH and NiFe LDH@EBP, and commercial RuO_2_, and **(D)** EIS curves for EBP, NiFe-LDH and NiFe LDH@EBP, and commercial RuO_2_.

Based on the aforementioned analysis, NiFe LDH@EBP was prepared using a simple bath sonication method to modify NiFe-LDH on the EBP surface by electrostatic adsorption and P-O valence bonding. To analyze the catalytic performance of NiFe LDH@EBP-catalyzed OER, LSV curves were collected in 1 M KOH aqueous solution. Pristine BP at a current density of 10 mA cm^−2^ is shown in Figure 5A. After stripping, the overpotential of EBP at mA·cm^−2^ was reduced to ∼400 mV as more active sites were exposed after liquid phase stripping. After loading NiFe-LDH, the NiFe LDH@EBP hybrid catalyst exhibited a very low overpotential of ∼240 mV, which was 30 mV lower than NiFe-LDH and also lower than commercial RuO_2_. In addition, the effect of the feed weight ratio of NiFe-LDH to EBP on the OER activity was also investigated, as shown in Figure 5A. From the LSV curves of NiFe LDH@EBP with different feed weight ratios of NiFe-LDH to EBP, NiFe LDH@EBP (1:2) showed the best OER activity. As the weight ratio of NiFe-LDH to EBP increases to 1:4 and 1:8, the OER activity of NiFe LDH@EBP decreases significantly. In fact, the intrinsic electrocatalytic OER activity of EBP is lower than that of NiFe-LDH, resulting in the decay of OER activity at large amounts of EBP. However, NiFe LDH@EBP (1:2) requires only a very low overpotential of about 240 mV to obtain a current density of 10 mA cm^−2^, which is about 63 mV lower than that of NiFe LDH (1:1). Thus, the synergistic interaction between NiFe-LDH and EBP effectively improves the catalytic activity, which mainly comes from the improved charge transport by the P-O covalent bond formed within NiFe LDH@EBP. In addition, the electrostatic assembly within NiFe LDH@EBP effectively avoids the agglomeration of NiFe-LDH and EBP during ultrasound-assisted co-assembly, which facilitates the exposure of the active site. (Wang and O’Hare, 2012; Jiang et al., 2016; Zhao et al., 2017).

As shown in Figure 5C, the Tafel slope was determined to better understand the catalytic kinetics of different catalysts. The Tafel slope of NiFe LDH@EBP (1:2) is ∼81 mV⋅dec^−1^ which is lower than that of BP (185 mV⋅dec^−1^), EBP (120 mV⋅dec^−1^), NiFe-LDH (120 mV⋅dec^−1^), and commercial RuO_2_ (99 mV⋅dec^−1^), implying the fast kinetic OER process. Typically, the catalytic processes for a typical OER start from the formation of OH* from adsorbed water molecules, the decomposition of OH* to O*, and the subsequent reaction of O* with another adsorbed H_2_O to form OOH*, and finally, the formation and release of O_2_. Therefore, different Tafel slopes reflect different rate determining steps (RDS) (Wang et al.; Song et al., 2020). Thus, for NiFe-LDH, the Tafel slope of 120 mV⋅dec^−1^ reflects RDS is the electron transfer process. After incorporating the EBP, accompanied by the reduction of the Tafel slope, the RDS turned into a mixed RDS involving the first electron transfer step and second step with an electro-transfer number of ∼0.5. Figure 5D shows the electrochemical impedance spectroscopy (EIS) curves collected at 1.5 V (vs. RHE) in a 1 M KOH electrolyte with an amplitude of 100 kHz-0.01 Hz. By fitting with the equivalent circuit, the R_ct_ for EBP, NiFe-LDH, and NiFe LDH@EBP are 4.8, 3.7, and 3.1 Ω, respectively. Therefrom, the NiFe LDH@EBP shows the smallest R_ct_ which is close to that of RuO_2_ (2.9 Ω). The co-assembly of NiFe-LDH and EBP via electrostatic adsorption and P-O valence bonding accelerated the electron transfer, and the large specific surface area of EBP also enabled the fast mass transfer (Chen et al., 2020).

**FIGURE 6:**
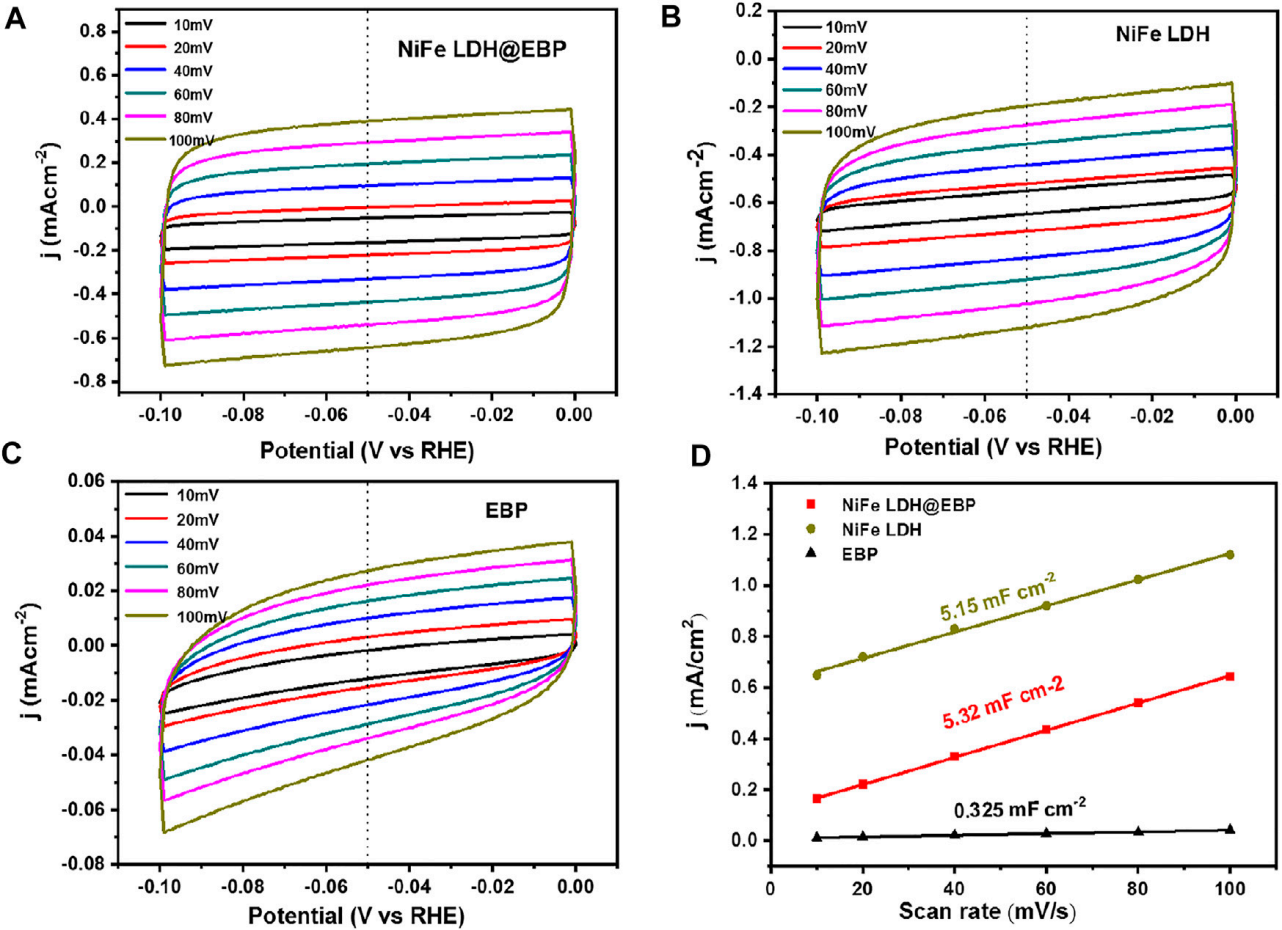
CV curves for **(A)** NiFe LDH@EBP, **(B)**NiFe-LDH, and **(C)**EBP at various scan rates for OER, and **(D)** double-layer capacitances (C_dl_) of NiFe LDH@EBP, NiFe-LDH, and EBP.

**FIGURE 7:**
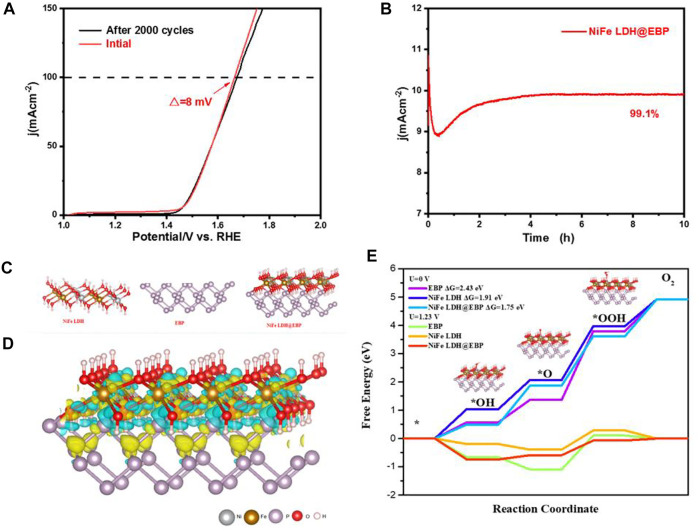
**(A)** LSV curves for NiFe LDH@EBP before and after the test of 2,000 CV cycles, **(B)** chronoamperometry curves of NiFe LDH@EBP at a current density of 10 mA cm^−2^, **(C)** optimized configurations of BP and NiFe-LDH heterointerfaces through P-O bonding, and **(D)** differential charge density of NiFe LDH@BP. The yellow and green contours represent the charge accumulation and depletion, respectively. The isosurface plotted is 0.003 eV/Å^−1^, and **(E)** energy profiles of the OER steps on BP, NiFe, LDH, and NiFe LDH@BP surfaces.

To better evaluate the electrocatalytic OER activity of NiFe LDH@EBP, the electrocatalytic active surface areas (ECSAs) were determined based on electrochemical double-layer capacitances (C_dl_) tested *via* CV scans at different scan rates of 10–100 mV/s in the potential region of 1.1–1.2 V (vs. RHE). As shown in Figures 6A–C, the comparison of NiFe LDH@EBP, NiFe, LDH, and EBP with different scan rates of 10–100 mV/s in the Faradaic potential range of 1.1–1.2 V relative to standard reversible hydrogen electrodes to scan. As shown in Figure 6D, the C_dl_ (5.32 μF⋅cm^−2^) of NiFe LDH@EBP is significantly higher than those of NiFe LDH (5.15 μF⋅cm^−2^) and EBP (0.325 μF⋅cm^−2^), suggesting that more active surface area is generated in NiFe LDH@EBP. The main reason is that in the process of the sonication-assisted assembly, the NiFe-LDH was exfoliated into thin NiFe-LDH nanoflakes which facilitates the exposure of a large number of active sites. Further, the catalytic stability of NiFe LDH@EBP was also investigated (Hao et al., 2019). The LSV curves of NiFe LDH@EBP (Figure 7A) before and after 2,000 cycles nearly overlap. There is about 8 mV of decay of potential at a high current density of 100 mA cm^−2^. Meanwhile, as shown in Figure 7B, the chronoamperometry tests of the NiFe LDH@EBP hybrid catalyst were also conducted. From the i-t curves, the current density first decreased, then increased, and finally remained stable, which may be attributed to O_2_ bubbles on the surface of NiFe LDH@EBP-based working electrode bubbles at the beginning and then gradually removed from the surface over time. Additionally, it is also possible that NiFe LDH@EBP experienced the process of activation. After 10 h of the i-t test, compared to the current density of 4 h, the current density of NiFe LDH@EBP catalyst could still maintain 99.1%, suggesting that the NiFe LDH@EBP also has good long-term catalytic durability toward OER.

For understanding the synergistic effect of NiFe, LDH, and BP, the density functional theory (DFT) calculations were carried out. Figure 7C shows the optimized configurations of BP (020) and NiFe-LDH (003) heterointerfaces through P-O bonding. The charge redistribution on the interface between BP and NiFe LDH with different specific electronic structures is shown in Figure 7D. There is evident charge redistribution near the interface, and electrons flow from CoFe LDH to BP, suggesting the strong interaction between BP and NiFe-LDH which is consistent with the analysis of XPS spectra. Figure 7E illustrates the reaction pathway of OER and the free energy diagrams at equilibrium potentials of 0 and 1.23 V. At the applied potential of U = 0 V, the rate-determining step (RDS) is the formation of *OOH from *O with free energy bases of 2.43, 1.91, and 1.75 eV for CoFe LDH, BP, and NiFe LDH@BP, respectively. The relatively low free energy of NiFe LDH-BP proves that BP and CoFe LDH are combined through P-O bonding effectively reducing the O-O formation energy and facilitates the thermodynamic formation of *O to *OOH. When a 1.23 V potential is applied, BP shows a more negative ΔG*O, which implies strong adsorption with *O, producing a significant upslope formation for the subsequent *OOH intermediate. For NiFe LDH@BP, the low ΔG values between the intermediates indicate a good balance between adsorption and desorption, which enhances the kinetics of the OER. Based on the aforementioned analysis, combined with the previous electrocatalytic activity analysis, the synergistic interaction between NiFe-LDH and EBP promotes the charge transfer kinetics and balances the adsorption/desorption performance of the reaction intermediates, which ultimately confers excellent OER electrocatalytic activity of NiFe LDH@EBP.

## Conclusion

In summary, the NiFe LDH@EBP with the exfoliated NiFe-LDH decorated on the surfaces of EBP was successfully prepared by facile sonication-assisted co-assembly method *via* electrostatic interaction and P-O bonding. When the feed weight ratio of NiFe-LDH to EBP equals 1:2, the obtained NiFe LDH@EBP exhibits a very low overpotential of ∼240 mV at a current density of 10 mA cm^−2^ which is significantly lower than those of individuals with the NiFe, LDH, and EBP. Meanwhile, the NiFe LDH@EBP also shows high electrocatalytic durability toward OER under alkaline conditions. Combined with DFT calculations, the combination of NiFe-LDH and EBP promotes charge transfer kinetics and balances the adsorption/desorption of reaction intermediates, ultimately imparting excellent OER electrocatalytic activity. This work will provide a promising strategy for the modification and functionalization of BP and expand the practical applications of BP toward electrochemical catalysis.

## Data Availability

The original contributions presented in the study are included in the article/Supplementary Material; further inquiries can be directed to the corresponding author.
